# Properties of healthcare teaming networks as a function of network construction algorithms

**DOI:** 10.1371/journal.pone.0175876

**Published:** 2017-04-20

**Authors:** Martin S. Zand, Melissa Trayhan, Samir A. Farooq, Christopher Fucile, Gourab Ghoshal, Robert J. White, Caroline M. Quill, Alexander Rosenberg, Hugo Serrano Barbosa, Kristen Bush, Hassan Chafi, Timothy Boudreau

**Affiliations:** 1 Rochester Center for Health Informatics, University of Rochester Medical Center, Rochester, NY, United States of America; 2 Clinical Translational Science Institute, University of Rochester Medical Center, Rochester, NY, United States of America; 3 Department of Medicine, Division of Nephrology, University of Rochester Medical Center, Rochester, NY, United States of America; 4 Department of Medicine, Division of Allergy, Immunology and Rheumatology, University of Rochester Medical Center, Rochester, NY, United States of America; 5 Department of Physics, University of Rochester, Rochester, NY, United States of America; 6 Department of Medicine, Division of Pulmonary and Critical Care Medicine, University of Rochester Medical Center, Rochester, NY, United States of America; 7 Oracle Labs, Belmont, CA, United States of America; Nankai University, CHINA

## Abstract

Network models of healthcare systems can be used to examine how providers collaborate, communicate, refer patients to each other, and to map how patients traverse the network of providers. Most healthcare service network models have been constructed from patient claims data, using billing claims to link a patient with a specific provider in time. The data sets can be quite large (10^6^–10^8^ individual claims per year), making standard methods for network construction computationally challenging and thus requiring the use of alternate construction algorithms. While these alternate methods have seen increasing use in generating healthcare networks, there is little to no literature comparing the differences in the structural properties of the generated networks, which as we demonstrate, can be dramatically different. To address this issue, we compared the properties of healthcare networks constructed using different algorithms from 2013 Medicare Part B outpatient claims data. Three different algorithms were compared: binning, sliding frame, and trace-route. Unipartite networks linking either providers or healthcare organizations by shared patients were built using each method. We find that each algorithm produced networks with substantially different topological properties, as reflected by numbers of edges, network density, assortativity, clustering coefficients and other structural measures. Provider networks adhered to a power law, while organization networks were best fit by a power law with exponential cutoff. Censoring networks to exclude edges with less than 11 shared patients, a common de-identification practice for healthcare network data, markedly reduced edge numbers and network density, and greatly altered measures of vertex prominence such as the betweenness centrality. Data analysis identified patterns in the distance patients travel between network providers, and a striking set of teaming relationships between providers in the Northeast United States and Florida, likely due to seasonal residence patterns of Medicare beneficiaries. We conclude that the choice of network construction algorithm is critical for healthcare network analysis, and discuss the implications of our findings for selecting the algorithm best suited to the type of analysis to be performed.

## Introduction

Network science can provide key insights into healthcare systems including patient referral patterns [1–12], provider communities associated with better healthcare outcomes, or specific drug prescribing patterns [13–15]. Network analysis is particularly useful for studying healthcare delivery by organizations (e.g. private practice groups and hospital networks) and providers (physicians, nurse practitioners, physical therapists, etc.). The research questions suited to network science methods typically fall into three categories: 1) network topology; 2) patient flow; and 3) provider clustering. Network topology questions include investigations of network structure and properties, such as the effect of the rules and constraints under which provider teams organize (i.e. referral bias, geographic proximity, insurance network restrictions)[16] or identifying providers with high levels of influence. In contrast, questions about network flow address patterns of patient movement, network capacity and dynamic instability (e.g. how influenza epidemics or hospital closures affect network capacity). Provider clustering can identify highly collaborative groups of providers associated with specific patient outcomes. Such work is crucial for identifying provider groups (e.g. communities, k-cliques or k-clans) with good outcomes for patients with complex conditions, such as cancer, heart failure or kidney disease [17–19].

All of these inquiries start by building a healthcare network model, with vertices representing providers or healthcare organizations, linked by edges representing the strength of the connection, generally the number of shared patients [1, 8, 11, 20]. Several types of network construction algorithms exist, each with specific applications. For example, matrix algebra methods are often used to construct social networks from moderate sized data sets, such as a provider-provider network [11]. In contrast, trace-route mapping algorithms are used to create network representations for the study of network flow (e.g. digital information, transportation, supply chains). These types of methods have been used to map the flow of information across the internet [21–23], through social networks [24–26], and metabolite flow in bacterial biochemical pathways [27]. However, studies of the strengths and weakness of different algorithms that might be used to construct healthcare networks are lacking in the literature.

The most basic algorithmic method of healthcare network construction is to find all the instances where a specific provider *x*_*i*_ sees a patient *y*_*i*_ at least once, create a large patient-by-provider table, and then transform it into a provider-provider network (PPN) with each vertex representing a provider and each weighted edge representing the number of shared patients between the two providers. This network construction method uses no temporal information about the direction of the provider-patient visits, but simply specifies the volume of shared patients over the sampling period. The resulting networks are well suited to identify provider teams or links between healthcare organizations, organization-organization networks (OON), that share large numbers of patients. In contrast, study of patient flow between providers requires building a network representation that captures the sequence of patient visits to providers, using algorithms that build networks based on the temporal ordering of provider visits. For example, adding up all of the visits where a patient goes from provider *P*_*i*_ → *P*_*j*_, with the time of the visits such that *t*_*i*_ ≤ *t*_*j*_ and doing this for all providers in a data set, yields such a flow network that describes how patients move through the healthcare provider network, and how they are linked. One such method (the sliding frame algorithm (see Methods *section*) has been used by the United States Center for Medicare Services to construct the annual United States Medicare Physician Referral Datasets [28–31], and to determine referral volumes from general practitioners to specialists [16, 32, 33].

Crucial to healthcare network analysis is selecting a network construction algorithm appropriate for the analytic goal, and understanding how the algorithm affects the results obtained from analysis. Despite the increasing use of network models to improve healthcare delivery and outcomes [1, 4, 5, 11, 12, 34–36], rigorous published reports comparing the properties of networks constructed with different algorithms are lacking. There is also little guidance addressing the choice algorithms for different types of analyses. Different methods are likely to result in networks with incongruent elements (e.g. numbers of vertices and edges), topology, and properties (e.g. vertex degree and centrality distributions, edge weights, communities identified). In addition, the relationship between network topology and meaning is complex and tightly linked. For example, do edges represent referrals, the act of a sending a patient to a provider for a specific consultation? What algorithms create networks best suited to identify teaming, the grouping of providers that share many common patients and collaborate on their care? Thus, the choice of network construction algorithm may have significant implications for network properties and inferred meaning of network topology.

The choice of network construction algorithm is also affected by the size of the data set and the computational complexity and memory required for the calculations [24, 37]. Healthcare networks are generally constructed from data with a simple data structure, each record containing the date, the provider’s unique identifier, type of event (e.g. visit, admission, lab test), and the organization of which the provider is a member (e.g. practice group, healthcare system). This data can then be linked to provider and patient demographic features and outcomes. The 100% sample Medicare Part B annual data sets contain ∼150–200 million individual claims from 800,000 providers for ∼25–40 million patients, giving ∼2.0 × 10^13^ data elements. This makes in-memory storage difficult, and network construction by conventional matrix dot product calculations computationally expensive [38, 39]. Algorithmic approaches, however, can provide an efficient and parallelizable implementation of network construction.

Motivated by these issues, we characterize the consequences of choosing particular network-generating algorithms on the study of healthcare delivery networks. In the following manuscript, we compare the network topology and properties of Medicare PPN and OON constructed from the same primary data set using three different algorithms, and discuss the implications of each method for healthcare network analysis.

## Materials and methods

### Human subjects protection

Research data were coded such that patients could not be identified directly, in compliance with the Department of Health and Human Services Regulations for the Protection of Human Subjects (45 CFR 46.101(b) (4)). The analysis presented here is compliant with Center for Medicare Services (CMS) current cell size suppression policy as well as all data exclusivity requirements contained in the CMS Limited Data Set Data Use Agreement. This project was approved by the University of Rochester Institutional Review Board under the “exempt” category.

### Data sources

Network construction algorithms were initially developed in PERL 5.22.1 using the CMS 2008-2010 Data Entrepreneurs’ Center for Medicare Services Outpatient Claims DE-SynPUF (DE-SynPUF) [40]. This file contains institutional outpatient annual claim information for a 5% sample of Medicare members’ outpatient Part B claims (i.e. 5% of all claims randomly sampled) spanning from 2008 to 2010. Each of the 15.8 million records in the DE-SynPUF file is a synthetic outpatient claim. The DE-SynPUF dataset is publicly available for developers to test algorithms [40].

After development, the algorithms were tested and validated on the 2013 Medicare Outpatient Claims 100% Limited Data Set (LDS) obtained from the Center for Medicare Services Research Assistance Data Center (ResDAC) [41]. These combined files contain over 160 million Medicare fee-for-service claims data submitted by all organization and individual outpatient healthcare service providers between January 1, 2013 through December 31, 2014, along with a unique claim identifier number, dates of service, and unique National Provider Identifier numbers (NPIs). All claims were included, as we wanted the analyses to be as close to the CMS criteria used for network construction, which can be found here: http://downloads.cms.gov/foia/physician_shared_patient_patterns_technical_requirements.pdf.

Provider information was abstracted from the National Plan and Provider Enumeration System (NPPES) data file [42] This file contains identifier information for all current and past United States licensed healthcare provider and organizations, each linked to a unique NPI number, and associated provider locations, demographics, and medical specialty information. We used the version from August, 2015, containing 4,763,891 NPI numbers of both organizations and individual providers. All NPI numbers were checked for validity using the Luhn algorithm [43]. Provider locations matched to a geo-coded NPPES downloadable file from July, 2014 by the North American Association of Central Cancer Registries (NAACCR) [44]. The file contains 4,180,737 NPI numbers and associated address, of which only 309 are lacking enough data to accurately geocode, and 179,614 are geocoded only at the zip code centroid level. Geocoding is to the second decimal point, giving a spatial resolution of 1.1 km (0.88 miles).

#### Data and algorithm availability

The Center for Medicare Services Outpatient Claims DE-SynPUF (DE-SynPUF) [40] test set is publicly available from the CMS web site. The full 2013 Medicare Part B Limited Data Set for Medicare claims can be obtained from the Center for Medicare Services. This data is bound by a privacy and limited distribution agreement, as well as HIPAA regulations, and thus cannot be made public with this manuscript. However, the files can be requested from the Center for Medicare Services by individual investigators and used to reproduce our findings. Release of the derived networks is also limited by Medicare requirements to remove nodes and edges where the total number of shared patients ≤11. This restriction is in place to prevent identification of individual patients based on a small number of visits to a unique combination of geographically identifiable providers [28]. Network construction algorithms are coded in PERL, can be found in S1 File and are released under a GPL 3.0 license. Censored networks are can be found in S2 File.

### Network construction algorithms

**Fig 1:**
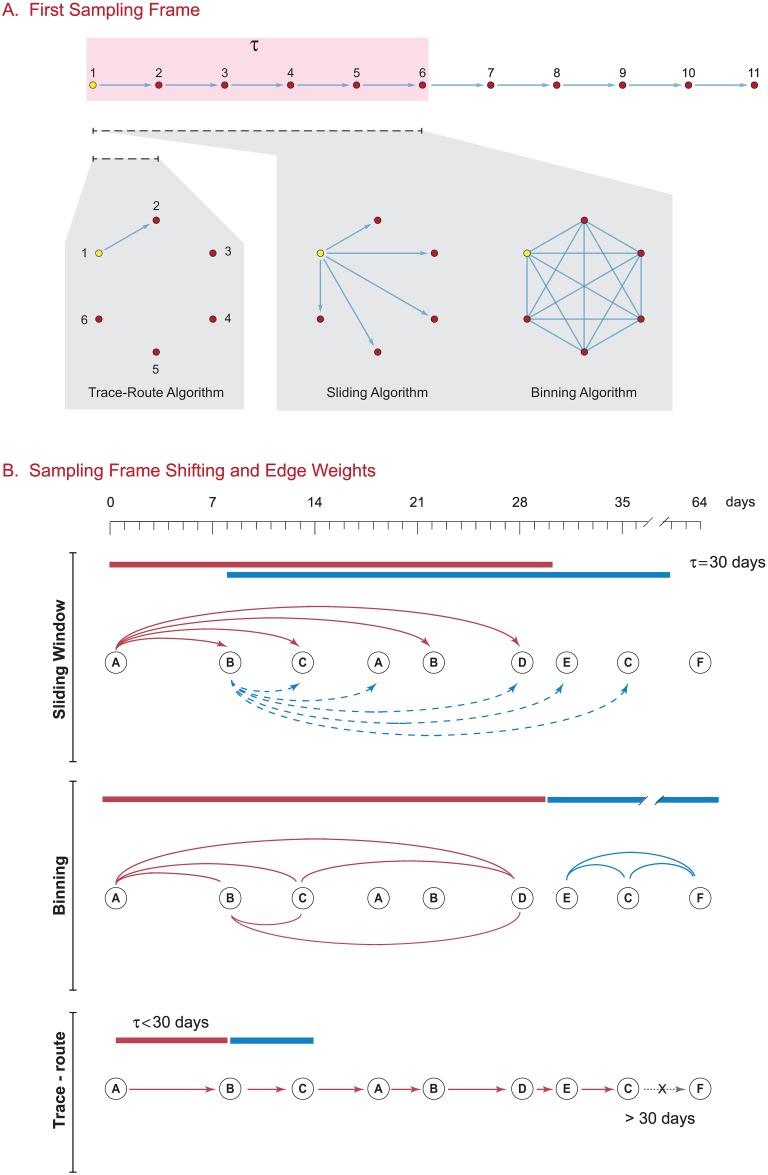
Edge construction algorithms for healthcare teaming networks. Each vertex represents a provider, with the index provider vertex in yellow. The collection of providers is for a single patient. (A) The brackets show how the time frame *τ* = 30 days is applied to a series of temporally ordered provider visits. The corresponding graphs show the edges that would be constructed between the provider vertices by each algorithm for that first iteration: a single directed edge for the trace-route algorithm, a set of directed edges (e.g. a star graph) for the sliding algorithm, and a complete graph with each vertex connected to all other vertices for the binning method. This process is repeated for each patient by shifting the sampling frame through the ordered visits for each patient. (B) Sampling frame shifting and edge weight construction. How each algorithm shifts the sampling frame *τ* through the series of provider visits is shown here, and the degree of shift for the next interval is shown by the new brackets. Please refer to the text for a discussion of edge weight calculations.

We constructed both provider and organization teaming graphs using three different algorithms, which we refer to as: (1) binning; (2) sliding frame; and (3) trace-route methods, adapting the terminology from Karimi and Holme (2013), who describe such frames in the context of dynamic networks [45]. The essential features of the algorithms are illustrated in Fig 1, with the subsequent mathematical description below and associated nomenclature listed in Table 1.

**Table 1:** Nomenclature.

Symbol	Definition
*v*_*i*_	Vertex (organization or provider) where *i* refers to identity of vertex type.
*k*_*i*_	Degree of vertex *i*
$e_{v_{k}\to v_{l}}^{j}$	Directed edge between vertex *v*_*k*_ and *v*_*l*_ for patient *j* (↔ when undirected).
*E*_*v*_*k*_ → *v*_*l*__	Edge between *v*_*k*_ and *v*_*l*_ over all patients.
$\omega_{v_{k}\to v_{l}}^{j}$	Edge weight of $e_{v_{k}\to v_{l}}^{j}$
Ω_*v*_*k*_ → *v*_*l*__	Edge weight of $\omega_{v_{k}\to v_{l}}^{j}$ over all patients
*V*, *E*, Ω, **P**, **C**	Respectively, sets of all vertices, edges, edge weights, patients, and claims
*t*_*i*_	Temporal instance of vertex *i*
*τ*	Censoring time frame interval (days)

#### Common algorithm features

All three algorithms selected data with a temporal visit proximity frame, only counting graph edges if the provider visits were within *t*_visit_ ≤ *τ* days of each other, where *τ* is the frame interval. For each method described below, let **C** be the collection of all Medicare claims such that *c*_*i*_(*p*, *v*, *t*) where *p* is the patient, *v* is the provider, and *t*_*i*_ the time of the patient visit for *i* = {1,2,3, ..*s*} where *s* is the number of claims. For all three algorithms, we consider all the claims for each patient *p*_*j*_ in the claims set **C**, where *j* = {1,2,3, ..*a*} and *a* is the number of individual patients. Each claim records the time of the patient visit to one provider. Claims are grouped by patient, and then sorted in ascending temporal order. The subsequent steps differ by algorithm, and are described below.

#### Binning network construction algorithm

The binning algorithm creates non-directed provider-provider network graphs. It is essentially an algorithmic implementation of the unipartite projection of a bipartite adjacency matrix [11, 12], with the potential advantage (for very large claims data sets) of not requiring in-memory matrix dot products for network creation. For the binning method, we start with the subsets of claims for each individual patient *p*_*j*_, where *j* = {1,2,3, ..*a*} and *a* is the number of individual patients. Iterating over each patient, we consider all the claims in each set and create edges such that:

$$e_{v_{k}↔v_{l}}^{j}\text{ if }\{\begin{array}{l} \left|t_{l}-t_{k}\right|\leq \tau \;\\ v_{k}\neq v_{l} \end{array}$$ (1)

where *t*_*i*_ refers to the time ordered instance of a particular claim (*i* being the identity of the provider). The total number of edges between vertices is

$$\begin{array}{c} E_{v_{k}↔v_{l}}=\sum_{j=1}^{a}e_{v_{k}↔v_{l}}^{j} \end{array}$$ (2)

where *t*_*i*_ refers to the time ordered instance of a particular claim (*i* being the identity of the provider). For individual patient edge weights within *τ*, only the first interaction for any given provider-provider pair and patient *p*_*j*_ is counted. For example, if $e_{v_{k}\to v_{l}}^{j}$ occurs 4 times within the frame *τ*, the weight is only counted once, or succinctly

$$\omega_{v_{k}\to v_{l}}^{j}=1\;\text{if}\;e_{v_{k}↔v_{l}}^{j}\neq 0.$$ (3)

The final edge weights **Ω** are calculated in a similar way as in the total edges by summing over all patients thus

$$\begin{array}{c} \Omega_{v_{k}↔v_{l}}=\sum_{j=1}^{a}\omega_{v_{k}↔v_{l}}^{j} \end{array}$$ (4)

#### Sliding frame network construction algorithm

Time directed network construction algorithms are designed to capture information contained in the temporal relationship of provider visits and used to build directed unipartite graphs. We first describe the *sliding frame algorithm*, one algorithm of this class. The sliding frame algorithm is similar to the current algorithm used to create the publicly available Medicare physician shared patient data sets available from the Center for Medicare Services website [29, 46].

In this setting, two providers are connected with a *directed* edge if two claims for visits with the same patient occur within time *τ* when claims are sorted by increasing order of time. That is:

$$e_{v_{k}\to v_{l}}\;\text{ if }\left\{ \begin{array}{l} 0<t_{l}-t_{k}\leq \tau \;\\ v_{k}\neq v_{l} \end{array}\right.$$ (5)

The requirement for *v*_*k*_ ≠ *v*_*l*_ excludes self-looping edges (e.g. sequential visits to the same provider). Edge weights are assigned as in the binning method described above, can be be weighted by incidence within the frame such that $\omega_{v_{k}\to v_{l}}^{p_{j}}$ is the number of occurrences of *e*_*v*_*k*_ → *v*_*l*__ for patient *p*_*j*_ within all frames *τ*. The final edge weight *Ω*_*v*_*k*_ → *v*_*l*__ within the entire graph of all patients **P** is calculated by

$$\begin{array}{c} \Omega_{v_{k}\to v_{l}}=\sum_{j=1}^{a}\omega_{v_{k}\to v_{l}}^{p_{j}} \end{array}$$ (6)

where *a* is the number of unique patients in **P**. If edges are counted more than once for a patient, *Ω* represents the edge weight of shared patients. In contrast, if edges are counted each time they occur between $\omega_{v_{k}\to v_{l}}^{p_{j}}$, they represent the total number of visits between providers of shared patients.

We refer to this method as the *sliding frame algorithm* due to the sequential scanning for relationships within the frame period *τ*. Some have proposed that this algorithm structure captures the urgency of patient “referrals” between providers, for example when *τ* = 30, where patients are directed by one provider to receive care from a second provider for an urgent medical issue [29].

Once the edges are created for each for patient, edge instances are tallied to obtain the overall edge weights for the entire network. Claims-weighted edges have the value of the total number of claims for patients shared by two providers summed over all shared patients. In contrast, patient-weighted edges are the sum of the number shared patients between two providers irrespective of the number of claims. The resulting provider-provider network graphs are weighted and directed.

#### Trace-route network construction algorithm

The trace-route algorithm is similar to that used to create a map of the internet, and traces the route of a patient through temporally sequential provider visits. The edges reflect provider-provider connections by sequential patient visits, and the edge weights are the rates of patient flow from provider to provider through the network for the period *τ*. The edge creation conditions can be specified by:

$$\begin{array}{c} e_{v_{k}\to v_{l}}\;\text{ if }\left\{ \begin{array}{l} \text{0 < }t_{l}-t_{k}\leq \tau \;\\ c_{k}\text{ and }c_{l}\text{ are strictly sequential claims} \end{array}\right.\; \end{array}$$ (7)

In contrast to other methods, self-loops are permitted such that *e*_*v*_*k*_ → *v*_*k*__ can be counted as an edge. Self-loop structures are common in strict temporally sequential claims data and reflect the case where an individual returns for successive visits to the same provider to address an ongoing condition or follow up after a procedure. Calculation of edge weights is the same as the sliding frame algorithm as noted above in Eq (4).

### Network comparison

Network comparisons were performed using standard network metrics in *Mathematica 10.4.1* or in Oracle PGX (see below). The definition of most metrics can be found in the excellent review by Newman [47]. Network metrics used in this manuscript included:

- *Component enumeration*: We enumerated the total number of vertices and edges within each network, and the largest connected component (*lco*) [47]. These correspond to the total number of unique providers or organizations, and the connections via shared patients between them.
- *Network diameter (d)* was calculated using Oracle PGX software with a longest shortest geodesic distance between vertices within the largest network component [47, 48]. The geodesic distance *d*_*ij*_ between any two vertices (*i*, *j*) is defined as the length of the shortest path between them. The network diameter *d* is defined as max *d*_*ij*_ ∀ (*i*, *j*).
- *Network degree assortivity (r)* is defined by the degree assortativity coefficient which has the form:
  $$r=\frac{\sum_{ij}\left(A_{ij}-k_{i}k_{j}/2m\right)k_{i}k_{j}}{2m-\sum_{ij}\left(k_{i}\delta_{ij}-k_{i}k_{j}/2m\right)k_{i}k_{j}}.$$
  Here *m* is the total number of edges, **A** is the adjacency matrix encoding the connectivity structure, *k*_*i*_ refers to the degree of vertex *i*, and *δ*_*ij*_ is the Kronecker delta function [47, 49]. Assortativity is a measure of whether like vertices connect to like vertices (in this case those with similar degree). This measure (which is formally equivalent to the Pearson correlation coefficient) lies in the range −1 ≤ *r* ≤ 1, with negative values associated with disassortative mixing (i.e high degree vertices more often connected to low degree vertices) and positive values with assortative mixing (i.e. similar degree vertices more often connected to each other). In many networks (e.g. social networks), vertices tend to be connected to others with similar degree values [50].
- *Network reciprocity (ρ)* is defined as the fraction of reciprocal edges over all edges in a directed graph, where *v*_*i*_ → *v*_*j*_ and *v*_*j*_ → *v*_*i*_ constitute a reciprocal pair [51]. In a directed graph, this provides a measure of how many bidirectional connections there are in a network. A low reciprocity in a directed healthcare network may suggest that patients only flow in one direction between two healthcare organizations *v*_*i*_ → *v*_*j*_, without minimal reciprocal flow, such as in hospice care referrals for terminally ill patients. In an undirected network, reciprocity is trivially 1 for all pairs of vertices by definition.
- *Global clustering coefficient (C) or transitivity* We can quantify the level of transitivity in a network as follows. If *u* knows *v* and *v* knows *w*, then we have a path *uvw* of two edges in the network. If *u* also knows *w*, we say that the path is closed–it forms a loop of length three, or a triangle, in the network. In the social network jargon, *u*, *v*, and *w* are said to form a closed triad. We define the clustering coefficient to be the fraction of paths of length two in the network that are closed. That is, we count all paths of length two, and we count how many of them are closed, and we divide the second number by the first to get a clustering coefficient *C* that lies in the range from zero to one [47, 52]. A high clustering coefficient in our networks can result when most providers are connected to other providers within the network, for example in a group practice that shares patients between providers.
- *Network density (D)* is calculated as *dm*/*n*(*n* − 1) where *n* is the number of vertices, *m* the number of edges, and *d* = 1 if the graph is directed or *d* = 2 if the graph is undirected [25, 53]. Network density provides a measure of how tightly connected elements of the network graph are, a ratio expressing the number of actual edges between vertices to the number of possible edges if the network were a complete graph (e.g. all vertices are connected to all other vertices). This gives a measure of how interconnected the entire set of healthcare providers or organizations are. For geographic networks at the city or regional level, network density may be quite high, but we might expect a low network density for the country as a whole as vertices based in different cities tend to be sparse. Providers on either cost are not likely to share many patients and therefore not be connected by edges.
- *Largest component size (lco)* is the number of vertices in the largest connected graph component [47, 54]. Some graphs may have several components (e.g. groups of edges) that are discontinuous, containing no common connecting edges. This is a measure of network fragmentation, for example when the *lco* is a small fraction of the total vertex count. In highly connected networks, the *lco* is the dominant component containing the vast majority of vertices.
- *Betweenness centrality (C_β_)* is calculated for an individual vertex *v*_*k*_ and is the number of shortest paths between all pairs of vertices that go through *v*_*k*_[47, 55]. For comparison between graphs, we also calculate (*C*′*_β_*), which is the normalized betweenness centrality such that *C*′*_β_* = *C_β_*(*N* − 1)(*N* − 2) for directed networks, and *C*′*_β_* = 2*C_β_*(*N* − 1)(*N* − 2) for undirected networks. A provider with a high (*C*_*β*_) value might be an oncologist, who receives referrals from many providers, but also refers patients to oncologic surgeons, radiation oncologists, hospice care, and many other types of providers. If that oncologist leaves the network and is not replaced, flow of patients from the

### High performance and parallel computing environment

Analyses were run on BlueHive2, an IBM parallel cluster located at the Center for Integrated Research Computing of the University of Rochester. We generally used two compute nodes, each with 2 Intel Xeon E5-2695 v2 processors with 12 cores and 64 and 512 GB of physical memory. Network analysis was performed using Oracle Labs Parallel Graph Analytics (PGX) toolkit version 1.2.0 and Wolfram *Mathematica* version 11.0 parallel computing and graph analysis functions.

## Results

Our focus here is the comparison of topology and properties of the healthcare network graphs built using three algorithmic methods: (1) a sliding temporal frame algorithm similar to that currently used to construct Medicare networks by the Center for Medicare Services [28, 29], (2) a temporal binning method which captures all possible relationships within a given time span (e.g. creates a complete graph of all providers who saw the patient), and (3) a trace-route algorithm [21, 22] that builds networks based on sequential sequence of provider visits. We have deliberately used networks generated from the Medicare Part B 2013 Outpatient Claims Limited Data Set, comprised of over 160 million claims, as opposed to a smaller data set. Our motivations were to (1) describe the differences in the topology of very large healthcare networks created by structurally different algorithms, (2) to investigate how these methods differ when used to study significant, real-world data sets, and (3) to examine the implications of algorithm design.

### Comparison of graph metrics

We first compared topological properties of network graphs constructed from the 2013 Medicare Part B Claims Data with the sliding frame, binning, and trace-route algorithms (Table 2). For this comparison, we used network graphs with *τ* = 365 days. Medicare Part B Claims data files contain insurance claims for all outpatient Medicare encounters in the United States over the course of a year. They do not contain charges for medications or hospitalizations. Provider vertices are individual providers that provided and billed for care during the data set period of 2013. Organization vertices represent provision of outpatient care by an organization. In addition, providers are generally associated with or belong to organizations (e.g. a group practice), and each claim generally contains both a provider and their associated organization NPI number. Because provider-provider networks (PPN) and organization-organization networks (OON) may have different network topologies and properties, and to separate the organization and provider dependencies of vertices, we constructed and analyzed separate networks for PPN and OON.

**Table 2:** Comparison of characteristics for patient co-care networks generated by different algorithms with *τ* = 365 days.

Metric	Provider-Provider Networks			Organization-Organization Networks		
	Sliding	Binning^‡^	Trace-route	Sliding	Binning^‡^	Trace-route
Edges (E)	89,377,290	65,287,590	40,077,297	3,282,133	2,233,601	2,014,859
Edge Type	Directed	Undirected	Directed	Directed	Undirected	Directed
Vertices (V)	811,784	811,784	814,917	40,749	40,749	40,768
*E*_*loop*_/*E* ^†^	-	-	0.411	-	-	0.122
*V*_*loop*_/*V* ^†^	-	-	0.938	-	-	0.943
*d*	51	29	89	6	13	10
*r*	0.05534	0.06985	0.02521	0.14768	0.16542	0.15915
*ρ*	0.56975	1.0	0.77929	0.86637	1.0	0.97295
*C*	0.28097	1.0	0.21598	0.53721	1.0	0.57809
*D*	0.00014	0.00010	0.00005	0.00198	0.00135	0.00119
*lco*	811,099	811,099	810,952	40,749	40,749	40,749
Max. V degree.	19,320	12,836	10,857	8,905	5,248	6,485
Mean V deg.	126.4	12.13	67.34	7.982	2.057	3.364
Max. E weight.	75,985	3,128	22,166	376,808	32,039	472,774
Mean. E weight.	7.982	2.057	3.364	126.4	12.13	67.34

*d*: network diameter, *r*: assortivity, *ρ*: reciprocity, *C*: global clustering coefficient, *D*: network density, *lco*: number of nodes in the largest component.

^‡^Metrics for undirected graph

^†^Algorithm explicitly excludes self-loops.

**Fig 2:**
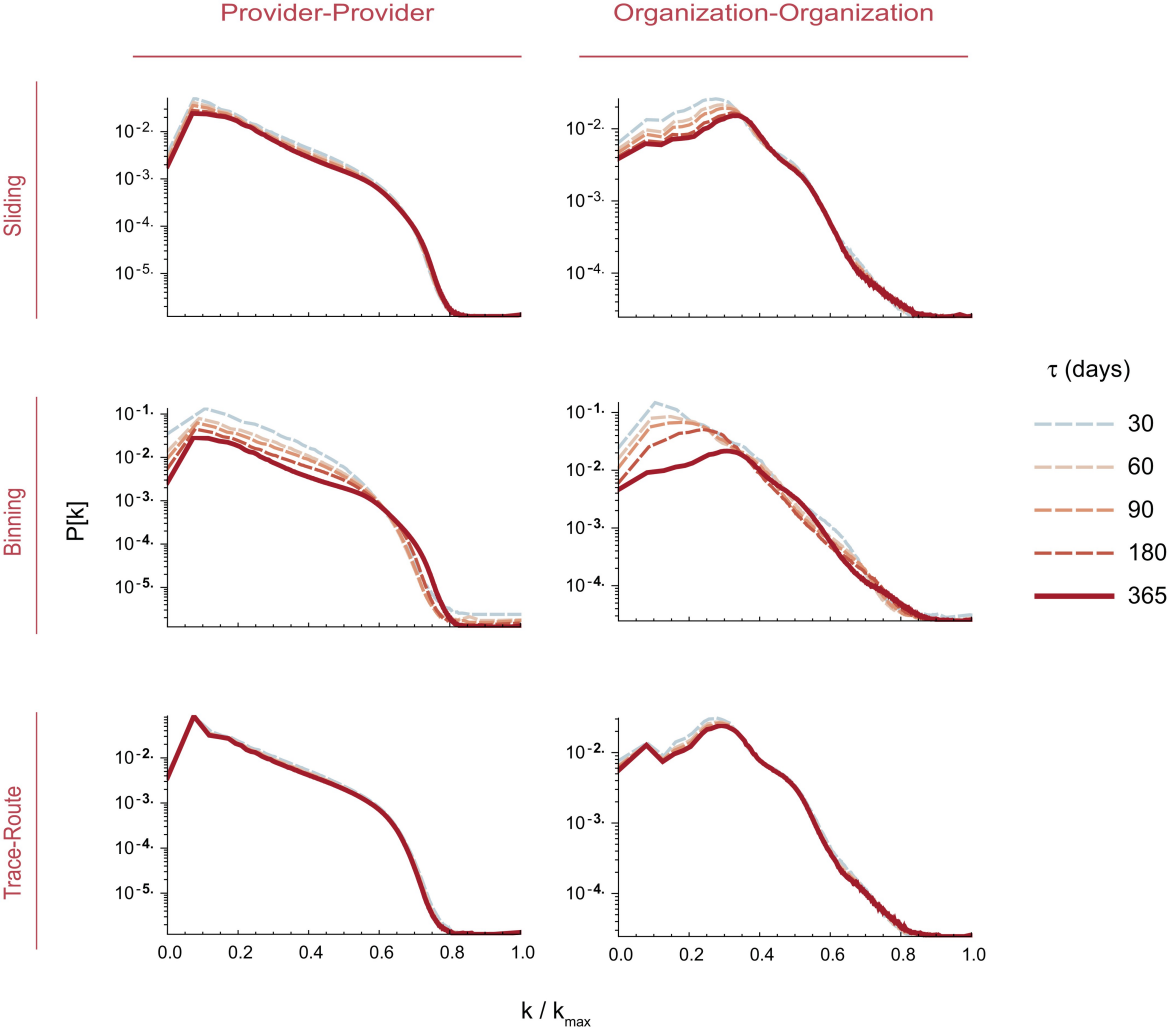
Network stability with different sampling frames. To assess network stability at various *τ*, we plotted normalized vertex degree (*k*/*k*_*max*_(*τ*)) versus *P*(*k*). Very small variations in the plots at *τ* = 30, 60, 90, 180 and 365 days indicate that network properties as a function of *k*/*k*_*max*_(*τ*) do not vary substantially.

All three algorithms yielded sparse networks (*D* < 0.00015; Table 2), with the trace-route method having the lowest density values(*D* = 0.00005). All algorithms also selected similar numbers of vertices *V*, with the trace-route algorithm producing modestly more vertices due to inclusion of degenerate self-loop edges (*v*_*i*_ → *v*_*i*_), representing sequential visits to the same provider. In contrast, the binning and trace-route algorithms resulted in PPN with markedly fewer edges (73% and 44% less respectively) compared with the sliding method, along with a higher graph density and and maximum vertex degree. The large components (*lco*) were essentially of identical size across all three methods, and for both PPN and OON graphs. In order to check the variation of the degree distribution *P*(*k*) with temporal frame *τ* we plot the rescaled degree *k*/*k*_*max*_(*τ*) in function of *P*(*k*) finding that for both the sliding frame and trace route algorithms, the vertex degree distribution properties are virtually identical for all *τ*. We do note, however, some variation for the binning method at low *k*/*k*_*max*_(*τ*) (Fig 2). While these results give confidence that the algorithms capture virtually identical sets of providers or organizations, the large variation in the number of edges *E* resulted in correspondingly large variations in network properties.

The binning algorithm generates non-directed graphs, and thus cannot be used to detect reciprocal events between providers with different edge weights (e.g. *v*_*k*_ → *v*_*l*_ coupled with *v*_*l*_ → *v*_*k*_), or to infer directionality of provider-provider interactions. However, the binning algorithm generates a complete provider graph for each patient, which makes it ideal for capturing complete “teaming” or for identifying larger communities of providers. In contrast, the sliding frame algorithm can also have multiple identical provider-provider or organization-organization edges (pairing weighted) for each patient, giving larger graph mean and maximum edge weights. This results from the sliding frame algorithm counting the same teaming interaction multiple times even if these are not sequential.

The trace-route algorithm yielded the smallest networks in terms of edge counts, primarily because edges are counted only when the visits between providers were sequential in time. The PPN created with the trace-route algorithm had a high fraction of edges that were self-loops (e.g. *v*_*i*_ → *v*_*i*_). Self-loops were present in 41.1% of all edges and 94% of all vertices in PPN and OON created with the trace-route algorithm. This reflects the common pattern where a patient will see the same provider in succession multiple times. Degenerate self-loops are not captured by the the binning or sliding frame algorithms. This is a key issue when creating networks to model patient flow through healthcare systems. If a large proportion of visits are sequential and to the same provider, algorithms that do not include degenerate self-loops cannot be used to accurately estimate network flow or capacity. The sliding window algorithm, similar to that used by the Center for Medicare Services to generate publicly available Medicare networks, does not have this feature.

**Fig 3:**
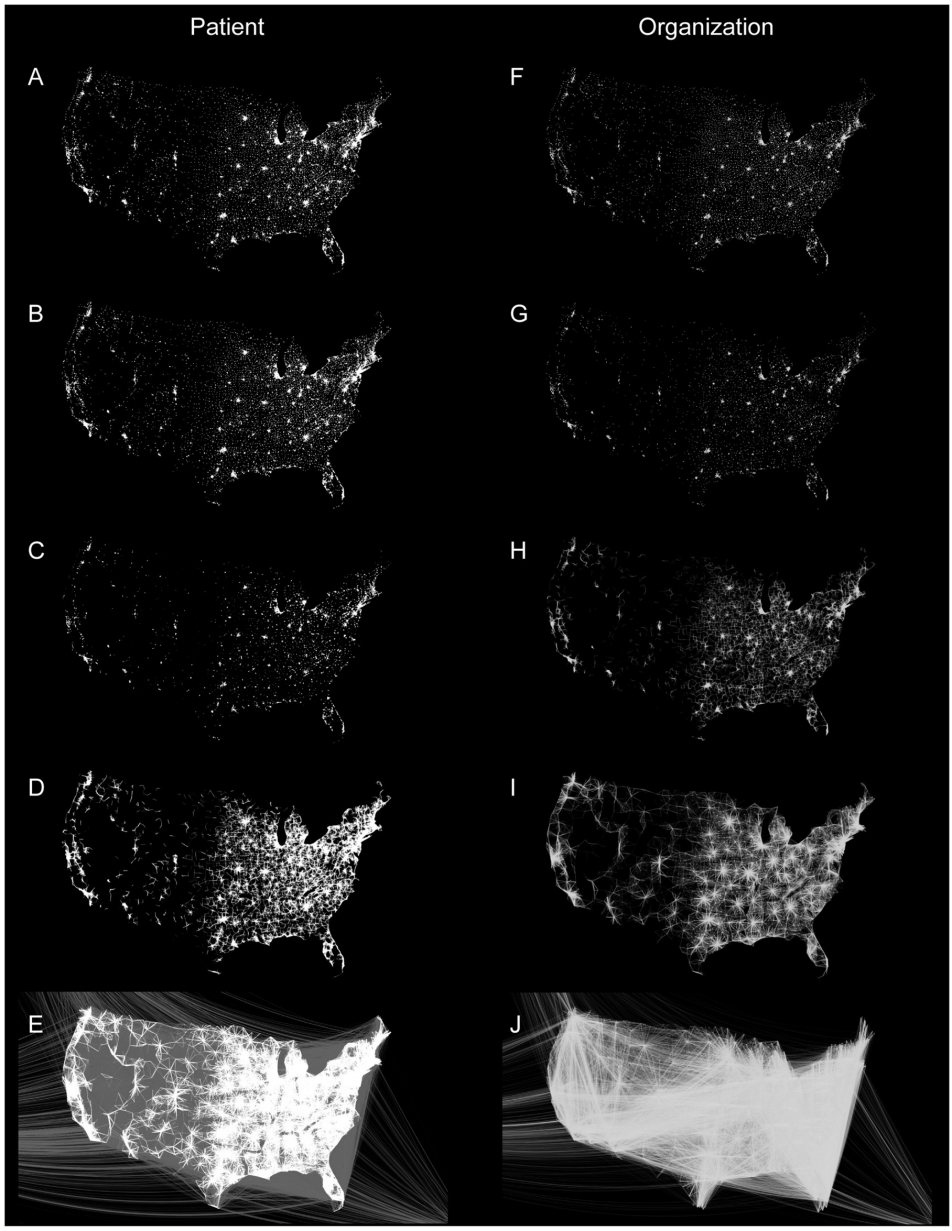
Geographic visualization of healthcare networks. Provider-provider (A-E) and Organization-Organization (F-J) healthcare networks for the Medicare Part B 2013 Limited data set created using the trace-route algorithm with a temporal frame *τ* = 365 days, and plotted using a geographic layout tied to provider location. The graphs are censored by removing weighted edges with a value of 1 (only a single shared patient) and excluding them from the visualization giving edge counts of 9,267,241 for PPN and 808,358 for the OON. Each PPN image contains ∼ 1–9 million edges, and each OON image contains ∼ 0.2-2 million edges. Images are binned by distance between providers: ≤ 1 mile (A,F), 20-40 miles (B,G), 100-200 miles (C,F), 800-1000 miles (D,G), 2000-4000 miles (E,H).

In order to uncover some of the spatial regularities associated with the constructed networks, we show a representative set in Fig 3, plotted with a geospatial layout. These networks were created with the trace-route algorithm, and each edge represents a sequential pair of visits between two providers. This is contrast to networks built with the sliding frame or binning algorithms, where edges do not represent sequential visits (i.e. two providers may have an edge despite the patient never having seen them in immediate succession). Given the rather large number of edges in the networks, for visualization purposes, we excluded edges with weights *Ω*_*j*_ = 1, which decreased the number of plotted edges for PPN by 76.9% and for OON by 59.9%. To further enhance the resolution of the visualizations, edges were sorted in ascending order by the distance between two providers that constituted the vertices of an edge, and then plotted in 16 separate network subsets of approximately by the geospatial distance between vertices (i.e. providers or organizations).

The supplemental figures contain high-resolution geospatial network plots (using identical thresholding) for PPN and OON created by the binning (S1 Fig), trace-route (S2 Fig), and sliding frame (S3 Fig) algorithms, respectively. These plots contain 16 figures for each combination of PPN or OON with each algorithm. Each of the 16 sub-plots contains a set of edges binned by the geographic-distance between provider or organization vertices in the edges (e.g. 2-4 miles, 4-10 miles, etc.), allowing a direct geospatial comparison of each method across plots.

There are several noteworthy features visible in the networks constructed by the trace-route method. The first is that the majority of edges appear to have very short distances (≤10 miles), suggesting that most Medicare patients have a set of providers in close proximity to each other. This seems likely a result of the Medicare population mix, individuals over 65 years of age, on dialysis, or with disabilities from complex medical conditions [56], as well as a general preference not to travel long distances from home for medical care. Another striking feature is the density of healthcare providers and organizations in the Eastern and Midwestern states, which correlates well with population density. In addition, note the spoke-and-hub appearance (Fig 3B, 3C, 3G and 3H) in both PPN and OON. This pattern appears to reflect travel between rural and urban areas. Importantly, these edges do not reflect hospitalizations, which are not contained in Medicare Part B claims data. Another notable feature is the presence of sequential visits between providers in the Northeast and the State of Florida (Fig 3D and 3F). These likely represent the “snowbirds”, patients who spend winters in Florida, and reflect a small proportion of Medicare patients for Florida providers (<1% of all visits to Florida providers). The migratory nature of this group is reflected in the north-to-south group of edges.

Furthermore, one can also see key differences between the PPN and OON in Fig 3. Notably the differences in edge counts are apparent in the visible edge densities, around ∼40 million for the PPN and ∼2 million for the OON. High resolution images for both the PPN and OON constructed using all three algorithms, censored and uncensored, are available in SI, S1–S3 Figs. Our preliminary investigations, thus indicate the valuable insights that one can glean through a geospatial representation of healthcare networks as it relates to patient journeys and flows.

### Censoring by edge weight markedly decreases network size

We next examined the effect of censoring edges with low edge weights. A key principle in public release of healthcare network data is to prevent identification of any individual patient, even within unipartite PPN or OON projections of bipartite networks where individual patients are not identified as vertices. An individual patient might be identified by a combination of unique providers they see where the edge weights between the majority of those providers is 1, and each provider can be identified by a geographic area. Such convergence to unicity (i.e. the ability to identify an individual from a unique combination of attributes) only requires a small number of attributes in very large data sets [57]. On consequence is that this may lead to fragmented networks, with many small sub-networks unconnected to the largest connected component. Medicare censors edges in publicly released provider teaming data sets by excluding those with weights <11 (“Presumed shared relationships based on claims for fewer than eleven distinct beneficiaries will be excluded from the report.”) [28]. Alternatively, others have suggested that censoring improves the signal-to-noise ratio for identifying strong provider-provider collaborations [12]. These studies have suggested that censoring at edge weights of 8-9 shared patients reflects provider self-identification of teaming partners [12].

To examine the effect that edge weight censoring has on the network properties, we compared uncensored and censored provider and organization networks created by each algorithm. We hypothesized that such censoring would lead to network fragmentation. We found that censoring resulted in a striking reduction in both nodes and edges (Table 3), as well as network density. This was most evident with respect to edges, where censoring for *Ω*_*v*_*j*_ → *v*_*k*__ ≤11 resulted in removal of more than 86-97% of edges for PPN, and more than 85% of edges for OON. To assess the effect of censoring level on network composition, we calculated vertex and edge counts for censoring thresholds from 1-11 shared patients (S3 Table). For PPN, censoring for edges with ≤ 11 patients for the trace-route, sliding frame, and binning methods reduced vertex numbers (49.9%, 31.2%, 70.8%) and edge numbers (2.3%, 2.1%, 13.8%) of the uncensored counts respectively. The largest reductions were for PPN provider pairs with only 1 shared patient for *τ* = 365. There modest relative decreases in both edges and vertices edge for weights for censoring thresholds between 8 and 11 patients. There is currently no current standard for labeling censored edges “noise”, with the true “signal” being the edges with *Ω*_*v*_*j*_ → *v*_*k*__ > *n*, where *n* is the threshold level. Thus, while censoring reduces network edges substantially, the level at which this contributes to improved community identification by improving signal-to-noise ratios, or results in loss of information with respect to patient mobility estimates, requires further validation with additional data that defines “true” and “noise” edges.

**Table 3:** Effect of edge weight censoring^‡^.

	Uncensored			Censored			$\frac{V_{Censored}}{V_{Uncensor}}$	$\frac{E_{Censored}}{E_{Uncensor}}$
	*lco*	*V*_*N*_	$\frac{V_{N}}{V}$	*lco*	*V*_*N*_	$\frac{V_{N}}{V}$		
**Provider**								
Sliding	810,099	685	<0.001	191,414	30,545	0.1376	0.273	0.017
Binning	810,099	685	<0.001	229,054	24,358	0.0961	0.312	0.045
Trace-route	810,952	3,963	0.005	87,533	228,444	0.7230	0.388	0.018
**Organization**								
Sliding	40,749	0	-	35,733	44	0.001	0.878	0.102
Binning	40,749	0	-	36,338	30	<0.001	0.892	0.092
Trace-route	40,749	19	<0.001	36,680	1,966	0.054	0.899	0.140

*lco*: vertices in largest component, *V*_*N*_: vertices not connected to largest component, *V*: total number of vertices, *V*_*Censored*_: total number of vertices in networks with censoring of edges with weights ≤11, *E*: total number of edges in uncensored networks; *E*_*Censored*_: total number of edges in networks with censoring of edges with weights *Ω*_*v*_*j*_ → *v*_*k*__ ≤11, *V*: total number of vertices in uncensored networks;

^‡^Metrics for undirected networks with *τ* = 365 days.

### Comparison of power-law characteristics of healthcare networks

**Fig 4:**
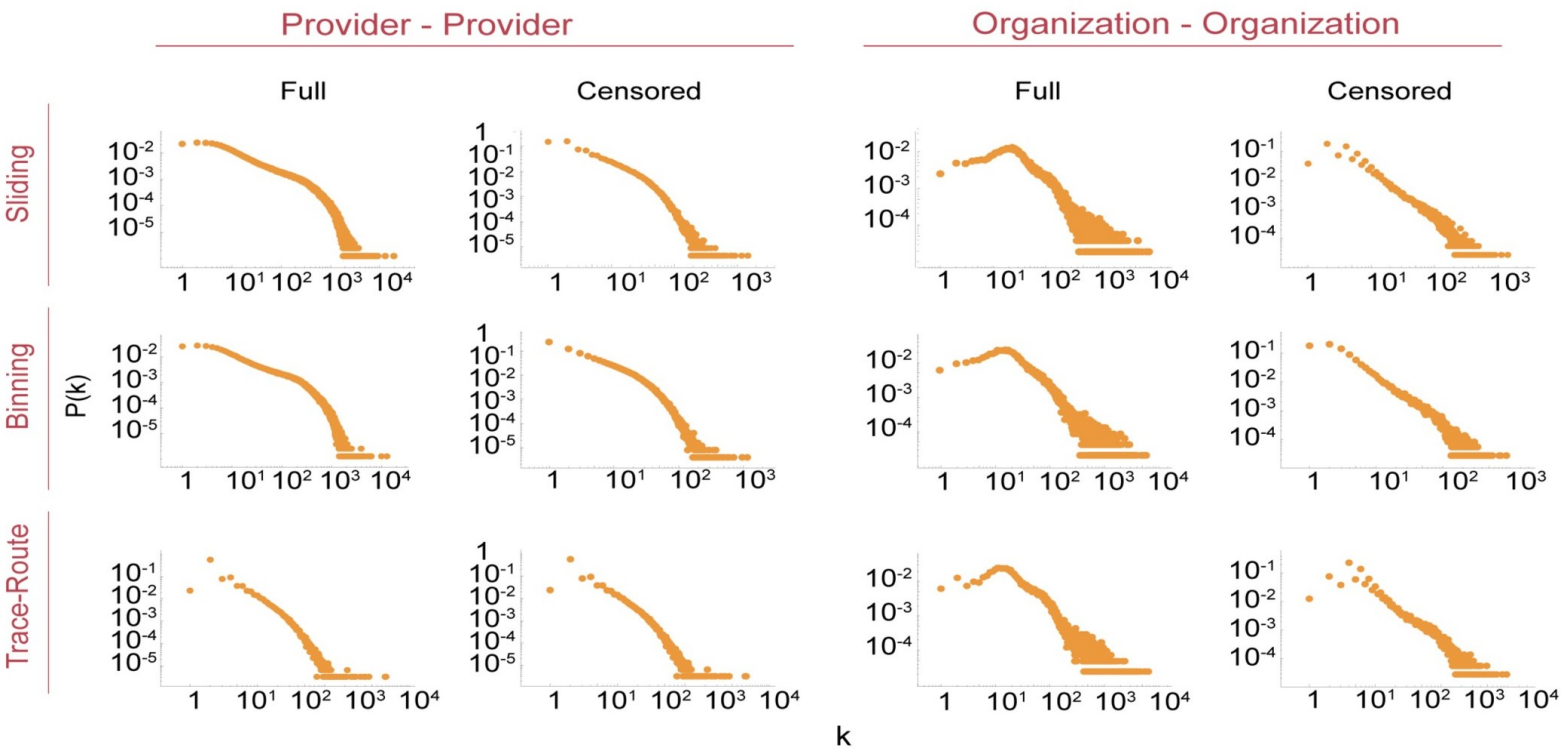
Network vertex degree distribution by algorithm for *τ* = 365 days.

We next tested networks generated by these methods to determine whether they were scale-free and adhered to a vertex-degree power law distribution. Many large and sparse networks are scale-free [26, 58–60], with power-law characteristics indicating a small number of central hubs with many edges, and a small-world topology [60]. Networks that can be described by power law distributions have distinct properties with implications for network formation and evolution [47, 58, 61]. In the case of healthcare networks, power law behavior may suggest how networks grow. For example a doctor in a new medical practice is likely to refer patients to other highly established providers with many connections, a phenomenon known as preferential attachment in graph theory [58]. For the PPN and OON built in this manuscript, vertex degrees *k* and their frequencies *P*(*k*) are shown in Fig 4. While both PPN or OON have heavy tailed distributions, neither appear to obey a strict power law distribution (e.g. *f*(*x*) ∝ *x*^ − *α*^). Interestingly, uncensored OON had a *P*(*k*) distribution similar to that found in relatively high density networks of internet discussion groups [62]. Censoring by edge weight *ω* < 11, however, decreased network density *D*, and altered the *P*(*k*) distributions in all networks.

We then used the method of Clauset et al. [60] to test PPN and OON network degree distributions for goodness of fit with a power law distribution, and to compare the fit with other discrete distributions (power law with exponential cutoff, exponential, log normal, Weibull, and Yule). If the power law had *p* > 0.1 then we accepted the null hypothesis that the data followed a power law distribution. To determine if the plausibility was significant we used the likelihood ratio (LR) to compare the fit with one of the other discrete heavy tailed distributions listed above [60]. If the LR values were negative with *p* < 0.05, we concluded that the network followed the alternative distribution being tested rather than the the power law distribution. The distribution with the most negative LR was selected as the best fit.

**Fig 5:**
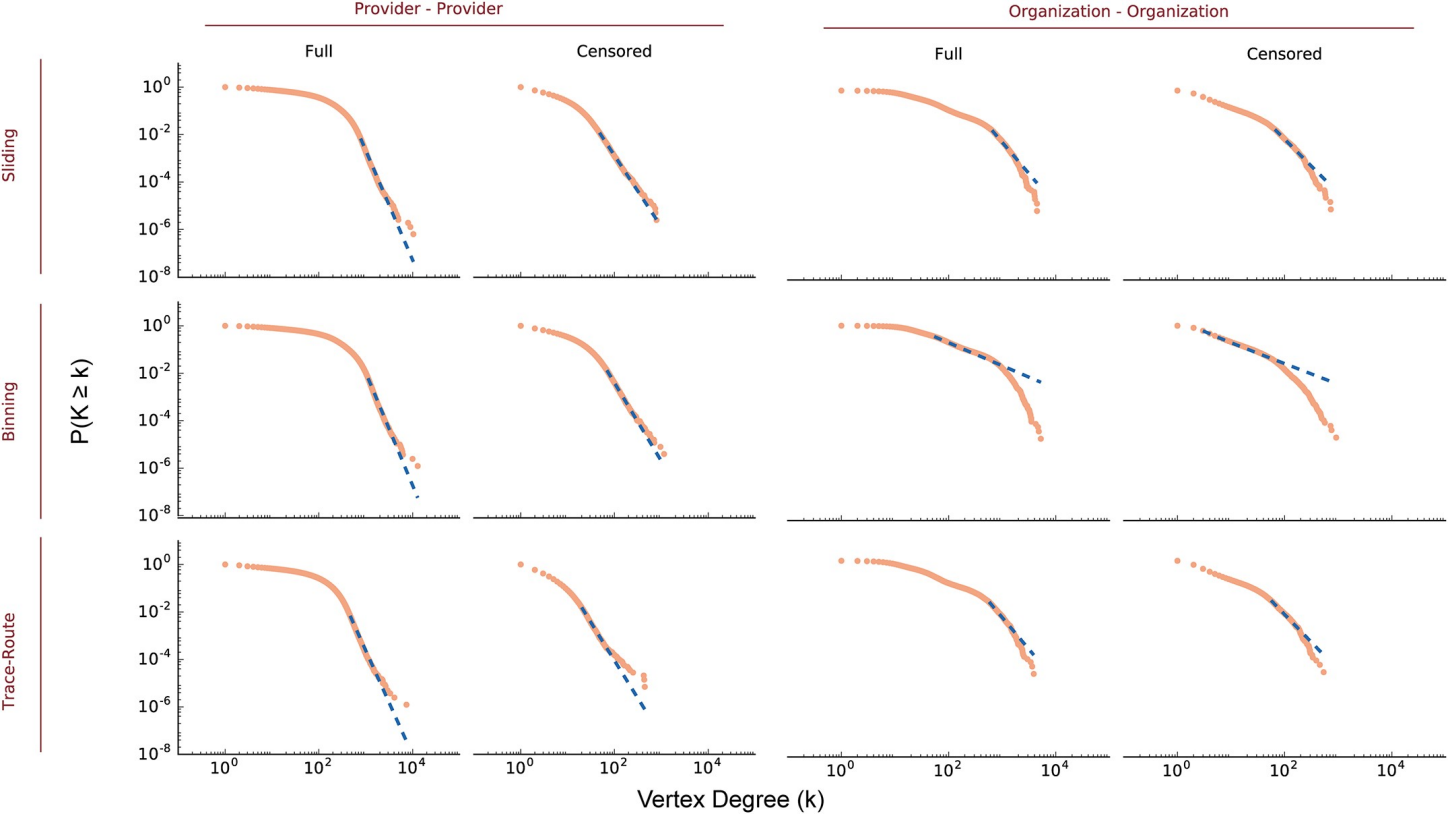
Healthcare networks power law with cutoff properties. We analyzed PPN and OON for adherence to power a law distribution starting from a minimum vertex degree, (*x*_*min*_), using the method of Clauset, et al [60] with *τ* = 365 days. The orange points are the CDF of the vertex degree, and the blue dashed line is the power law fit of the CDF given *x*_*min*_ and *α*.

In our analysis (Fig 5), all but one of the full and censored PPN adhered to the power law distribution with thresholding, that is beyond a value of *x*_*min*_, with statistical significance S2 Table. Goodness of fit testing for the Poisson distribution yielded likelihood ratio results that were approximately the order of magnitude of 5 (i.e. 10^5^), and hence insignificant. However, none of the other heavy tailed distributions fit the full PPN created with the sliding frame algorithm, and all had an LR>0.

In contrast to PPN, the best goodness of fit on the OON was the power law with exponential cutoff (PLEC; S2 Table). This distribution is often seen in network analysis of human mobility [61], reflecting an opportunity cost for traveling larger distances, or building networks that are geographically constrained by physical limits, such as power grids. Similarly, the spatial distributions of for-profit and public facilities obey a PLEC distribution, which is consistent with previously described models where there is a higher financial cost for locating facilities in areas of sparse population [59]. In the case of OON, the better fit to a PLEC distribution suggests that shared numbers of patients between healthcare organizations decay proportional to the distances between organization service areas. We hypothesize that this may be due to the structure of the data, where every Medicare Part B claim must have an organization listed, and >99% of the claims list both a provider and an organization. Thus OON may be considered a dimensionally reduced PPN, and we hypothesize that this may account for differences in fitting to power law distribution variants.

### Vertex centrality distribution varies by network construction algorithm

**Fig 6:**
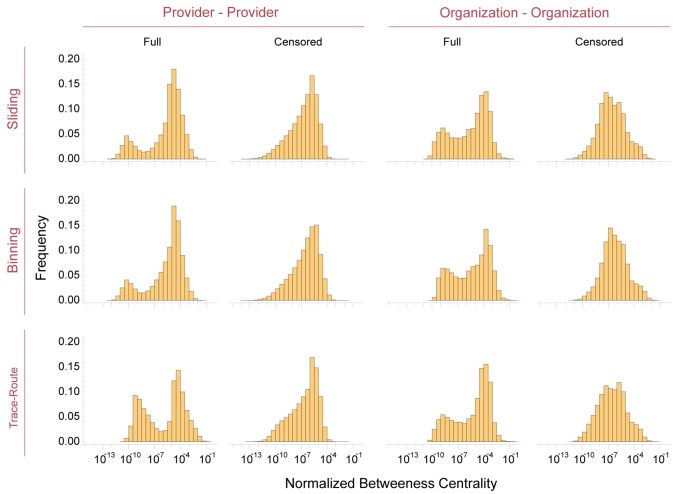
Betweenness centrality *C*′*_β_* of healthcare networks by algorithm for *τ* = 365 days betweenness centrality was calculated for all networks using the Oracle PGX algorithm. Results are displayed with algorithmic binning of *C*′*_β_* = *C^β^* / (*N* − 1)(*N* − 2) for directed graphs produced by the sliding frame and trace-route algorithms, and *C*′*_β_* = 2*C^β^* / (*N* − 1)(*N* − 2) for undirected networks produced by the binning algorithm. All plots are scaled in the y-axis to frequency, allowing direct comparison of centralities. Note that edge-weight censoring (excluding edges with Ω_*v*_*j*_ → *v*_*k*__ ≤ 11) markedly changes the centrality distribution of all networks.

We next analyzed differences in the centrality and connectivity of individual vertices (providers or organizations) between the networks generated by the three algorithms. Centrality metrics may be used to rank organizations by the proportion of shared patients with many other organizations, and can also be used to analyze healthcare service provision disparities or revenue potential. (Fig 6) Normalizing betweenness centrality (*C*′*_β_*—see Methods), and plotting the frequency rather than absolute distribution, allows direct comparison of all the networks despite their differing size and scales.

The *C*′*_β_* distributions of networks produced by the different algorithms are quite similar. The major difference is between full and censored networks. The addition of nodes that are only connected by edges with *Ω*_*v*_*j*_ → *v*_*k*__ ≤ 11 introduces a bimodal distribution of *C*′*_β_*, reflecting the low centrality of the previously censored nodes. This is consistent with the hypothesis that most of these providers or organizations are on the periphery of the network backbone, and are unlikely to create new connections between other providers or organizations that already have high *C*′*_β_* or *k*_*i*_ values. Another possibility is that the this phenomenon reflects variation in the proportions of total patients with Medicare insurance seen by providers. Some providers may see a large percentage of Medicare patients (e.g. nephrologists and geriatric medicine practitioners), while others may see only a small number of Medicare patients but a much higher proportion of patients with private insurance, leading to a bimodal distribution of *C*′*_β_*.

### Network variation by temporal sampling frame interval

**Fig 7:**
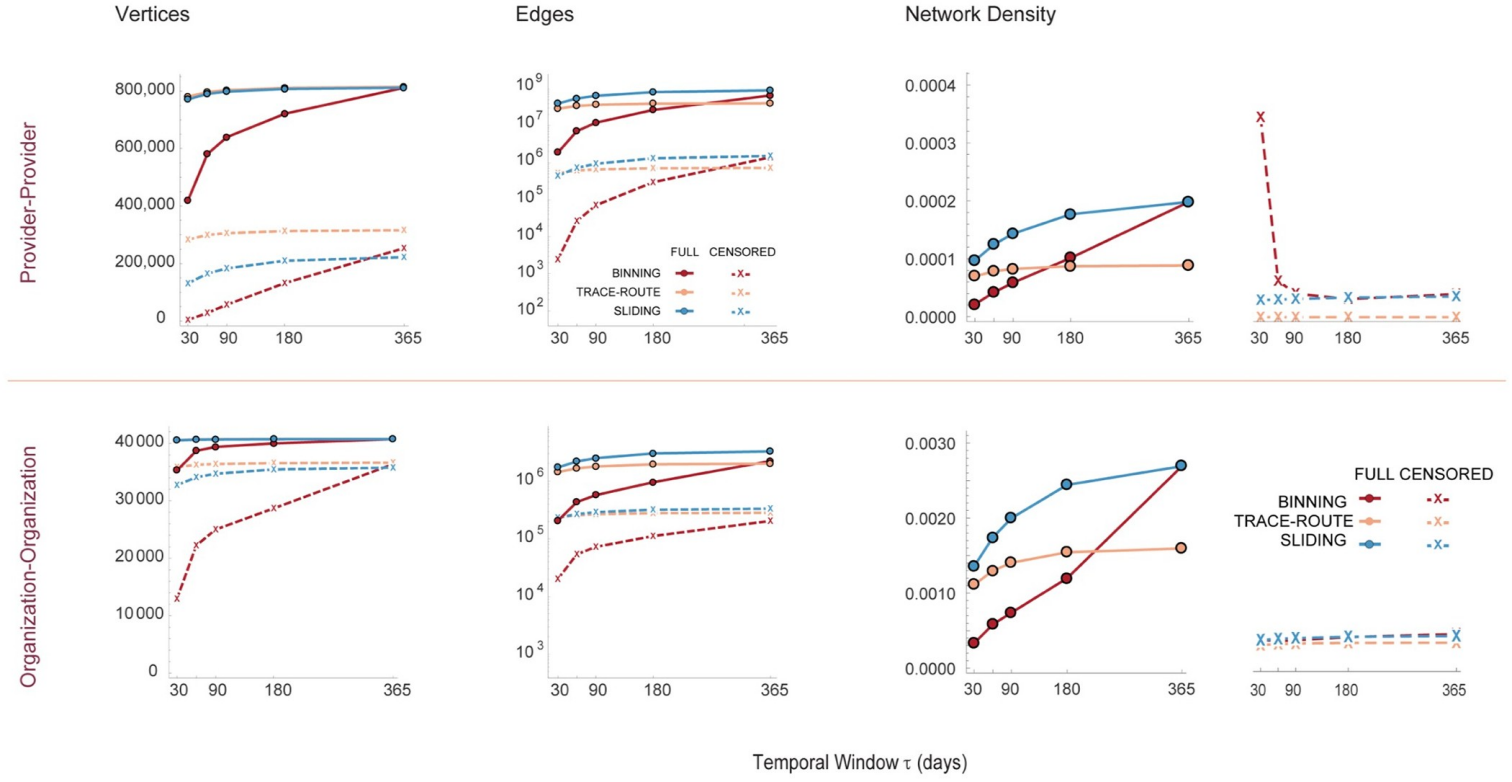
Network vertex counts, edge counts and density as a function of the sampling frame interval *τ*. Vertex counts, edge counts and network density plotted for provider and organization networks for the binning (red), trace-route (orange) and sliding frame (blue) algorithms for *τ* = 30, 60, 90, 180, and 365 days. Solid lines represent networks where vertices were included if the minimum edge weight > = 1, while dashed lines represent censoring where only edges with a minimum edge weight > = 11 are included. The latter is the current standard for aggregate provider network data release by the Center for Medicare Services so that individual patients cannot be identified by a unique combination of providers sharing only a single patient.

Healthcare networks are dynamic; the shared number or volume of patients between providers (e.g. edge weights) changes over time based on frequency of patient visits and, less frequently, as new providers are added to or leave the network. Of particular interest has been the number of patients shared between two providers during a specific time period. This measure might reflect the efficiency of patient flow through a healthcare system. For example, if the number of edges reflecting shared provider visits within 30 days is small, it might suggest that patients are unable to get urgent consultations in a timely manner. Alternatively, examining networks built from visits during a time frame *τ* can help determine if longer *τ* provide a fuller picture of the network (Fig 7). We found that over ≥ 98% of vertices and edges are captured with *τ* ≥ 90 days for both PPN and OON by the trace-route and sliding frame algorithms (see S1 Table). Considerable variation, however, occurs with the binning method, and the number of vertices or edges included converges on that of the binning and trace-route methods only as *τ* approaches 365 days. These findings suggest that the major topology of claims-derived healthcare networks can be captured by the trace-route and sliding frame algorithms with *τ* ≤ 180 days, while the binning method may not converge until *τ* ≥ 365 days.

### Variations in network community identification

One use of PPN is to identify highly collaborative teams or communities of providers. Such communities can arise from shared patients or membership in administrative organizations (e.g. Accountable Care Organizations, practice networks, or group practices). Provider teams can be identified by network community identification algorithms [26], as well as hierarchical or agglomerative clustering methods [63, 64]. Prior work on community detection has also used geographic or administrative regions to further refine the clustering constraints. The composition and number of communities identified will vary by method, and is a function of vertex connections via edges and edge weights [8, 9, 11, 12]. Thus, it is highly likely that networks built using different algorithms, when analyzed by the same community identification method, will yield different groups of providers. To test this hypothesis, we examined community assignments resulting from networks generated using the same data by each algorithm.

**Fig 8:**
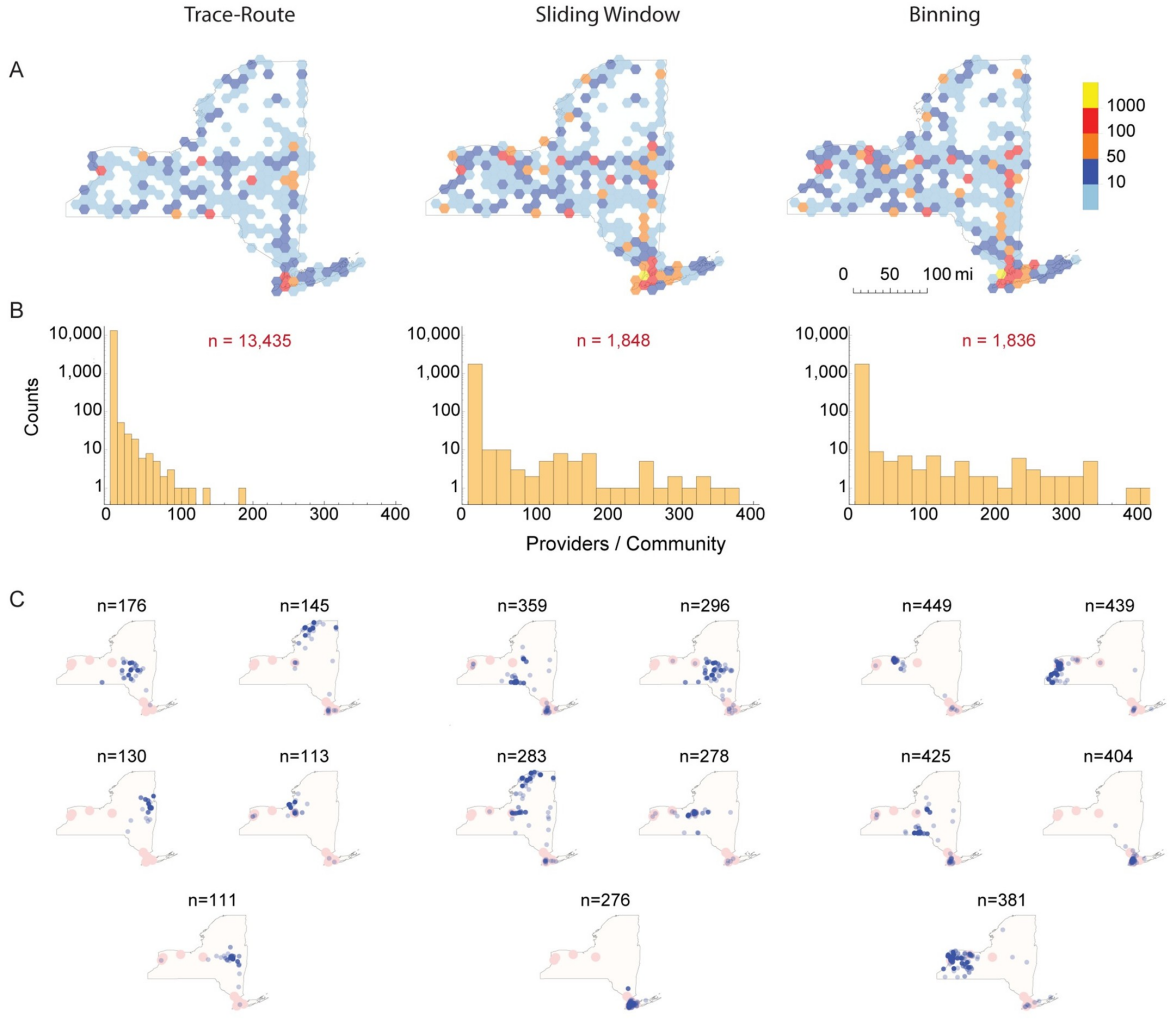
Variation in provider community identification. We analyzed undirected Provider-Provider networks constructed with the trace-route, sliding frame and binning algorithms for *τ* = 365 days, and censored for edge weights ≤ 11. Provider-Provider community teams identified for providers within NY State from each network using the Girvan-Newman modularity community finding algorithm [26] implemented in *Mathematica*. Each provider was assigned to only one community. (A) Provider densities. Hexagonal bins show the counts of providers that were members of any community within each geographic region color coded by range. Note the different geographic density patterns for each method. (B) Histogram of number of providers per community. Note the large number of communities (*n*) in each histogram, with the majority having only 2 providers. Community sizes, compositions and number differed between all 3 methods. (C) Shows the five largest communities identified in each network.

Fig 8 illustrates how networks built from the same data set using the trace-route, sliding frame, and binning methods yield different provider communities. For simplicity of comparison and computational efficiency, we selected only edges where both providers were located in NY State. A community is defined as a set of vertices (e.g. providers) who have a larger number of connections (e.g. shared patients) with each other than vertices outside the community. For this analysis, we started with PPN for *τ* = 365 days, and censored for edge weights ≤ 11. Communities were identified by the Girvan-Newman modularity community finding algorithm [26]. We found marked variation in the number, size, and composition of the resulting communities. Similar results were found when this procedure was applied to other states. Not only is the number and geographic distribution of providers belonging to communities different (Fig 8A and 8B), but the community size and geographic distribution ranked by number of providers also differs substantially. The community partitioning of the trace-route networks yielded a large number of small communities (97% with *n* ≤ 6) compared to the sliding frame and binning method networks (46% and 44% with *n* ≤ 6 respectively). The geographic location of the communities, when ranked from largest to smallest, also differed substantially (Fig 8C). This analysis highlights the significant differences seen in identifying provider communities when networks are built with different algorithms. For example, the trace route method has a lower representation of providers in New York City in the largest 5 communities than the binning or sliding frame algorithms.

## Discussion

Healthcare networks are commonly constructed from insurance claims data to study patient referrals, provider teaming and communication. They are used to identify network topology, patterns of provider association, and to test whether these correlate with healthcare outcomes or as part of comparative effectiveness research. The results of such analyses are increasingly used to shape healthcare services delivery and policy, with potential to impact millions of providers and patients, However, little work has been published addressing how network algorithm selection may effect network topology and analysis. Our results demonstrate that different algorithms will yield networks with different topologic properties, and analysis of these networks yeilds quite different results. These results support our conclusion that algorithms should be carefully selected based on the question being asked, the algorithmic approach used to define edges and edge weights, the sequence and frame used for temporal data, and the degree of edge weight censoring.

It is critical to select a network building algorithm designed to study the questions being asked, specifically network flow versus community identification. In the context of the work presented here, flow represents the aggregate mobility pattern of patients traveling between providers or organizations in the underlying network. The trace-route algorithm is specifically designed to focus on directed and sequential pair-wise interactions, including sequential visits to the same provider, reflecting almost exclusively the underlying mobility network [21, 22]. Trace-route networks are commonly used to model flow of packets through computer networks [23], vehicles through highways [65], and goods through supply chains [66]. In contrast, the sliding frame or binning algorithms are based on a computational approach designed to capture the emergence of abstract (and possibly hidden) collective structures, such as clusters and communities [21, 28, 29]. Importantly, these networks capture provider teaming and identify linked communities even if patients do not sequentially visit all providers within their team. To illustrate, imagine a patient visits providers A, B and C, and then travels to another state and visited providers D and E. The binning or sliding frame algorithms would create connections between *all* the five providers. This network would include clusters and sequential patient flow patterns that are absent from the original data (e.g. *A* → *C*, *B* → *D*, etc.) and do not exist from a mobility perspective. Such frame-based approaches are even more problematic when dealing with large scale healthcare networks, where less frequent, long range, geographic connections will form the backbone of the nationwide network. Thus, the choice of algorithmic approach should be considered by the problem at hand (e.g. community identification versus flow analysis).

Another important consideration in the interpretation of healthcare networks is the mistaken inference of causality. For example, both the trace-route and the sliding window methods use temporal ordering of claims. However, this simple temporal sequence and does not imply causality. For example, a patient may have been to their oncologist, then 5 days later to the Emergency Room, next had a yearly physical with their Family Practitioner, and then a follow up to the ER visit some days later. Here we are simply capturing that sequence. The oncologist may not have referred the patient to the ER five days later (e.g. after an automobile accident unrelated to breast cancer 5 year follow up), and the ER visit did not “cause” the physical. Similarly, we should be careful inferring that teams identified in network analysis imply deliberate and preferential teaming of specific providers. The choice of providers may be highly determined by external factors such as geography (e.g. there is only one abortion provider within 300 miles) or payment constraints (e.g. the patient’s insurance will only reimburse for visits with specialists within the insurance network). Thus, careful analysis and external validation are needed when using network structure to infer voluntary teaming for laudable goals (e.g. providing the best care for prostate cancer) or more sinister intent (e.g. insurance fraud or inappropriate narcotics prescriptions).

Another key finding of our work is the difference between the vertex degree distribution of organization and provider networks. Organization networks tended to follow a power law with exponential cutoff, while the provider networks followed a power law. This is likely due to geographic constraints. Medicare is administered at the state level, and providers are geographically based. This suggests that there may be a threshold that limits the value for *organizations* to extend their interactions (e.g. share patients), or for patients to obtain medical supplies or seek outpatient care, over long distances. Practically, this limits the vertex degree of healthcare organizations, and their regional associations. It is interesting that provider-provider relationships do not exhibit the same constraints, especially as our analysis indicates that most patients see providers located within a small radius of care. Providers in PPN tend to add connections exponentially such that new providers tend to link with established and well connected providers. In addition, patients may be willing to travel longer distances for certain specialty care (e.g. cancer therapy, organ transplantation), but unlikely to travel far for lab testing or medical equipment in OON. This suggests that the presence of underlying differences in the social, structural and economic motivations between providers and organizations, and that further work to better define these could be done comparing different healthcare systems (e.g. single versus multi-payer systems, differences in geographic constraints, etc.), and further detailed analysis using network segmentation by claim codes.

The level at which to censor patient weighted edges remains an active area of discussion in the literature, either to prevent patient identifiability or to improve the signal-to-noise when using clustering to identify provider teams [11, 12, 67]. Medicare uses a cutoff of 11 shared patients for censoring edges to prevent patient identifiability by a unique pattern of providers. Other work has used external validation via survey data to assess at what level of patient sharing pairs of providers would consider each other as collaborating [11], finding that a threshold of 9 shared patients correlated well with provider teaming identification [12]. Given the scope of our analysis, 4 − 8.5 x 10^7^ edges and 880,000 providers, such survey work would require statistically guided sub-sampling of provider pairs [11, 12]. Sensitivity analysis, assessing results of community identification at different censoring thresholds, may also provide guidance. Thus, an important area for further work is external validation of provider community identification under different levels of censoring.

As far as we are aware, this is the first comparative analysis of how network algorithm structure influences the topology of healthcare networks built using administrative claims data. Such inference of network topology based on analysis and comparisons of algorithm structure is common in the computer science and informatics literature [68–70]. Overall, our work makes two types of inferences: those based on examining and comparing algorithm structures, and those resulting from analysis of networks created by applying the algorithms. Our analysis suggests the use of trace-route type algorithms for creating networks intended to study time-ordered migration of patients from one provider to another. The trace-route algorithm structure shows that edge construction is constrained to sequential provider visits; this is intrinsic to the algorithm, and independent of the data used to build a network. Thus, by definition, trace route networks only contain edges that reflect sequential temporal ordering. In contrast, the binning and sliding window algorithms create additional edges that reflect possible, but not actual, temporally sequential patient visits. With respect to inferences drawn from analysis of the networks, censoring by edge weight dramatically reduces the number of network edges and alters network topology. Furthermore, depending on the type of analysis undertaken, mixing provider and organization vertices can be problematic given the dependencies between organizations and providers (e.g. provider connections may be highly dependent on organization membership), and the differences in network topology between PPN and OON.

Currently, the Center for Medicare Services has not released the data selection methods or algorithm code used to construct the deidentified and censored provider teaming networks, although the resulting networks are publicly available to researchers, policy makers, and businesses. This is a substantial issue for health services research rigor and reproducibility [71, 72], with implications for healthcare policy discussions. Without knowing the algorithm structure or performing sensitivity analyses, the dependencies of the resulting networks on claims filtering or network construction methods remain unknown. Together, these findings suggest that research rigor and reproducibility may be improved by building networks from Medicare claims data using verifiable algorithms, and analyzing the sensitivity of network topology to the censoring criteria. Future studies will be needed to address the issue of which algorithms are “optimal” for specific research questions (e.g. with respect to computational efficiency, community identification, and other measures).

Finally, our work suggests several other directions of inquiry, including comparisons of networks between states with different Medicare structures, detailed investigation geographic dependencies of community identification algorithms, analyses of provider team composition and outcomes, studies to identify provider migration to different geographic locations, and studies of patient flow through healthcare systems.

## Conclusion

The topology of healthcare networks constructed from claims data varies is a function of the algorithms used to construct them. Consequently, the analytic results obtained will vary accordingly, including network density, edge weights, vertex centrality measures, and community identification. The size and topology of healthcare networks built using administrative data is particularly sensitive to the effect of edge censoring, and the methods used to construct and weight edges. Sensitivity analysis of network topology as a function of these factors can aid in identifying these issues.

## Supporting information

S1 Fig Healthcare network plots created with the binning algorithm. Provider-provider and organization-organization network plots for *τ* = 365 days as discussed in the Results section. Networks are plotted using geospatial coordinates accurate to within 0.8 miles in the continental United States. Each sub-plot represents a range of distances for edges between providers or organizations to allow comparisons across methods. These are also available online: PPN (https://figshare.com/s/1054f48521ee7f75b715; doi: 10.6084/m9.figshare.3827220), OON (https://figshare.com/s/8e4c310b512d5c285c03; doi: 10.6084/m9.figshare.3827217).
(ZIP)

S2 Fig Healthcare network plots created with the trace-route algorithm. Provider-provider and organization-organization network plots for *τ* = 365 days as discussed in the Results section. These are also available online: PPN (https://figshare.com/s/ef453524484445b9f3e3; doi: 10.6084/m9.figshare.3827532), OON (https://figshare.com/s/eb15be0bed3fd0b71f82; doi: 10.6084/m9.figshare.3827520).
(ZIP)

S3 Fig Healthcare network plots created with the sliding window algorithm. Provider-provider and organization-organization network plots for *τ* = 365 days as discussed in the Results section. These are also available online: PPN (https://figshare.com/s/638bd98a64c59c620978; doi: 10.6084/m9.figshare.3827505), OON (https://figshare.com/s/7c68005ef9d19a2ab6b7; doi: 10.6084/m9.figshare.3827361).
(ZIP)

S4 Fig Healthcare community identification for NY state. Provider-provider communities with *n* >5 providers were identified in networks built for New York State providers only. These plots show all of the provider locations for each identified community. Major cities are identified in red. Figures are also available online: PPN (https://figshare.com/s/638bd98a64c59c620978; doi: 10.6084/m9.figshare.3827505).
(PDF)

S1 File Network construction algorithms. This file contains the sliding, trace-route and binning PERL algorithms for network construction, and are also available online at figshare.com. (https://figshare.com/s/4f926e28a36700fc20a5, doi: 10.6084/m9.figshare.3837717).
(ZIP)

S2 File Censored networks. This file contains the data used to construct all the censored networks. Censored network files are also available from figshare.com at (https://figshare.com/s/915649f63d8fe8b08c5e, doi: 10.6084/m9.figshare.3833943).
(ZIP)

S1 Table Table of graph metrics as a function of measurement frame *τ*. This table, in TSV format, contains all of the the metrics for 200 networks built using the binning, trace-route, and frame algorithms for providers or organizations, with or without censoring, directed or undirected edges, and edge weights reflecting shared patients, or total numbers of visits for the shared patients.
(CSV)

S2 Table Power law best-fit results for patient co-care networks with *τ* = 365 days. This table contains results for fitting of power law distributions to patient co-care networks.
(PDF)

S3 Table Edges and node fractional reduction as a function of thresholding by edge weight. This table has shows the fraction reduction in edges and nodes as a function of thresholding the edge weights.
(PDF)
