# Supplementary material for: Properties of healthcare teaming networks as a function of network construction algorithms
Source: PLoS One. 2017 Apr 20;12(4):e0175876. doi: 10.1371/journal.pone.0175876 (PMC5398561; doi:10.1371/journal.pone.0175876)

Binning Algorithm - Community Identification

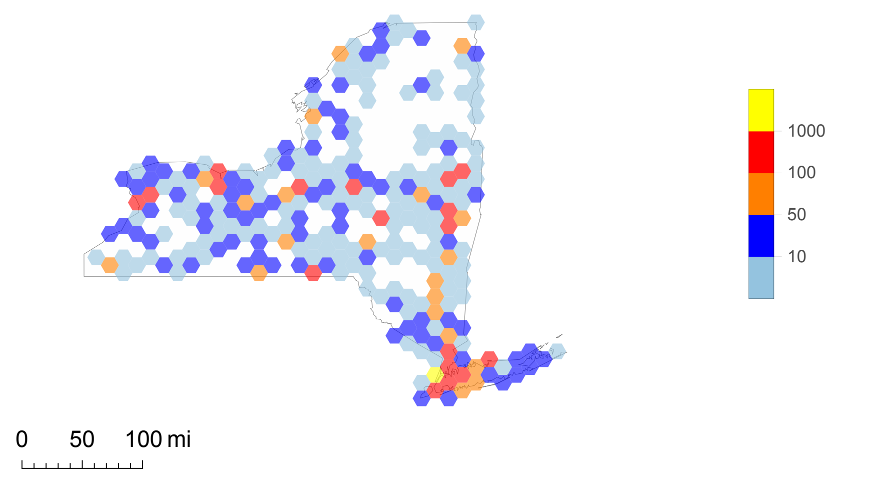

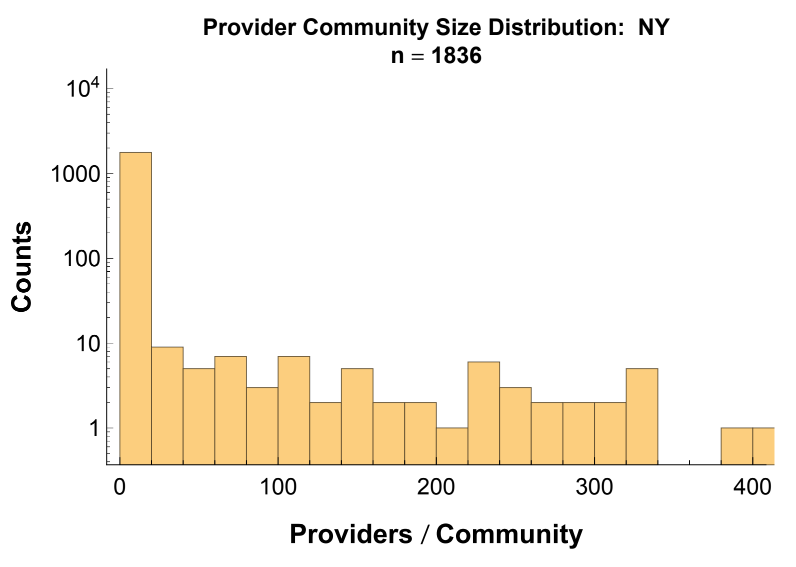

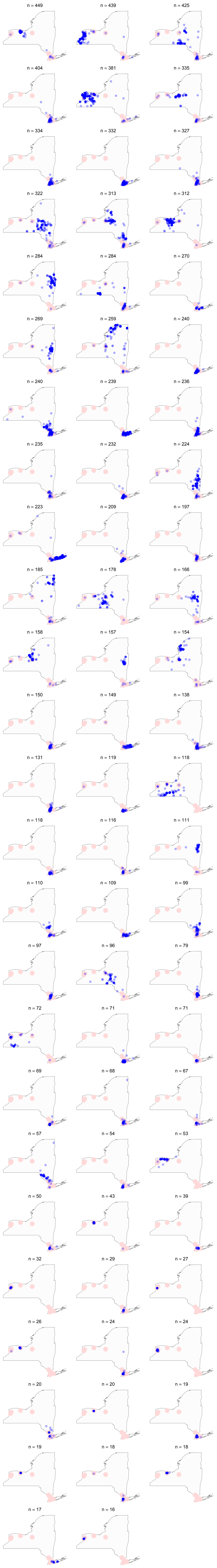

Sliding Frame Algorithm - Community Identification

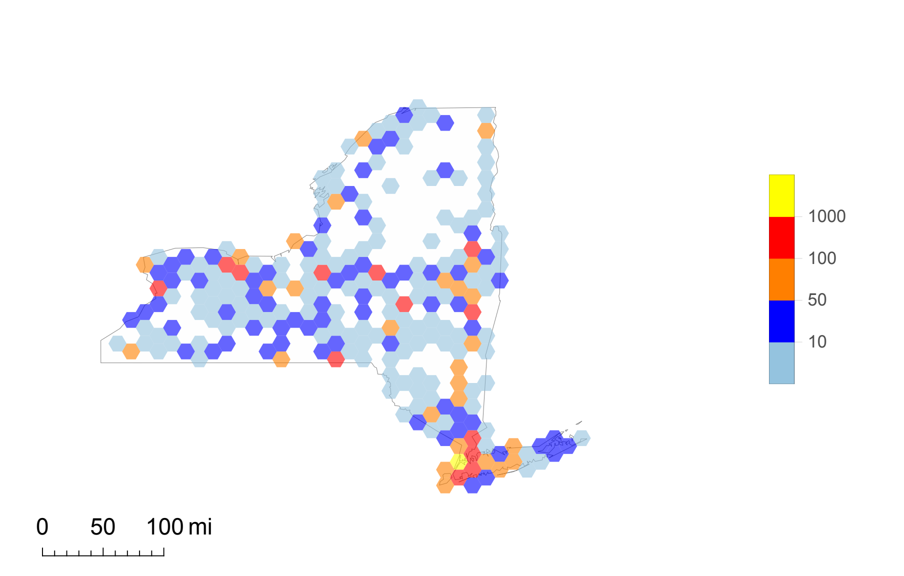

Provider Community Size Distribution: NY
n = 1836

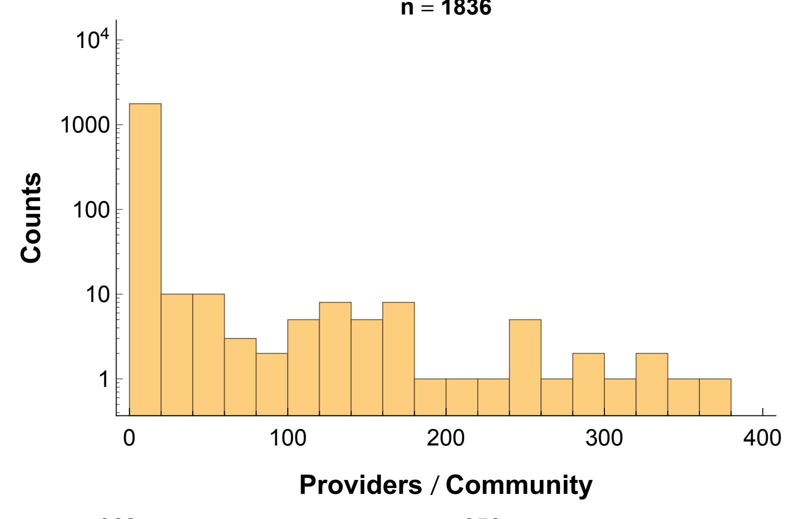

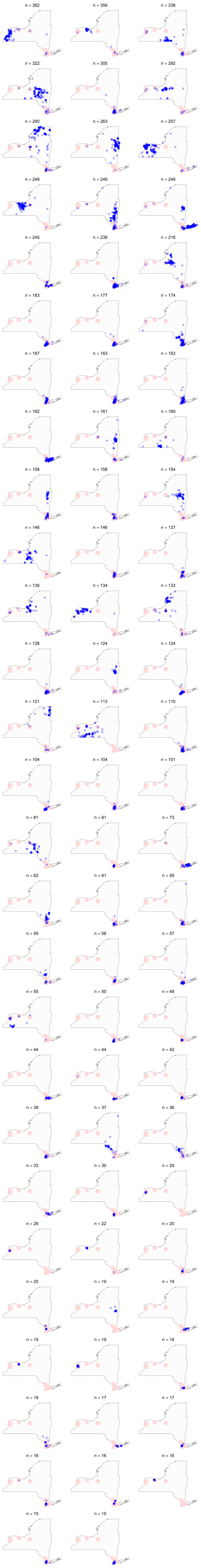

Trace-Route Algorithm - Community Identification

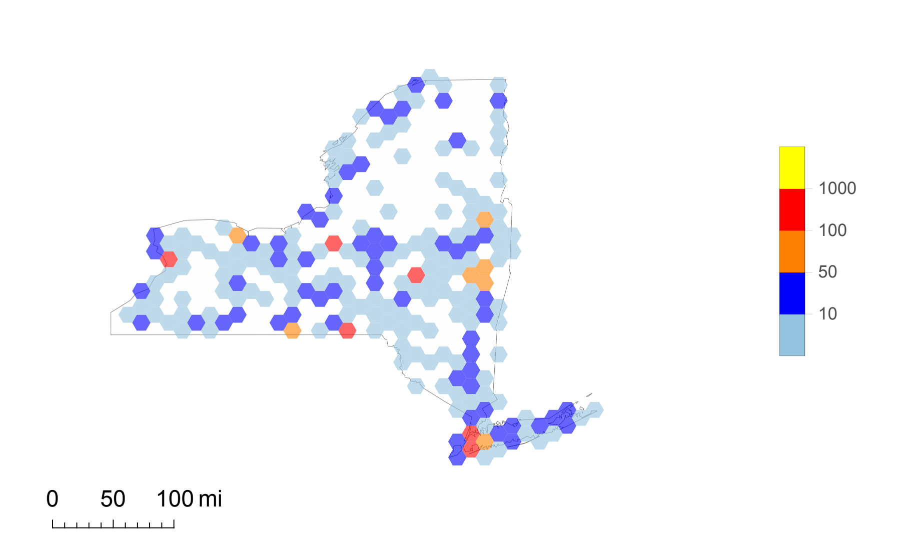

Provider Community Size Distribution: NY
n = 13469

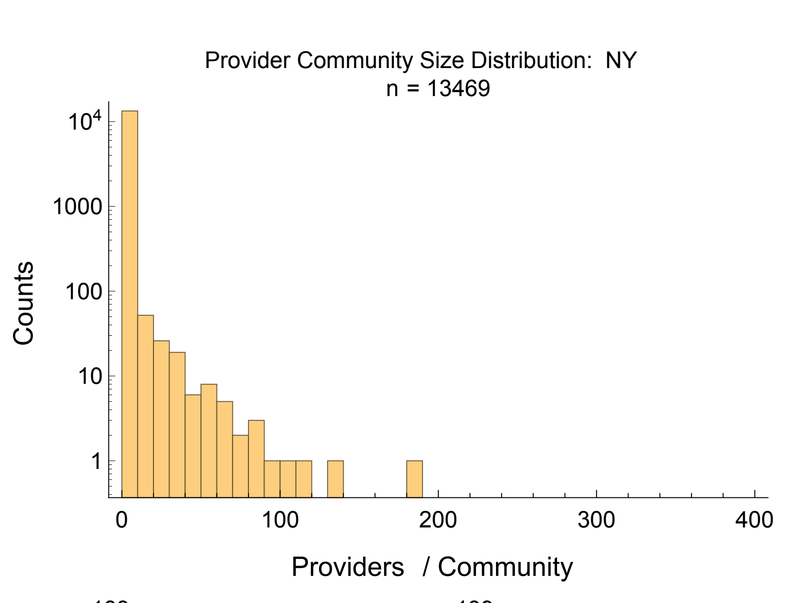

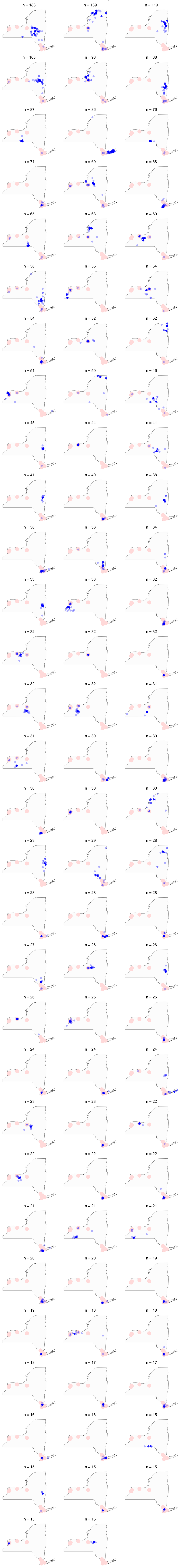

Supplement: S4 Fig — Provider-provider communities with n >5 providers were identified in networks built for New York State providers only. These plots show all of the provider locations for each identified community. Major cities are identified in red. Figures are also available online: PPN (https://figshare.com/s/638bd98a64c59c620978; doi: 10.6084/m9.figshare.3827505). (PDF) [file pone.0175876.s004.pdf]
