# Supplementary material for: Properties of healthcare teaming networks as a function of network construction algorithms
Source: PLoS One. 2017 Apr 20;12(4):e0175876. doi: 10.1371/journal.pone.0175876 (PMC5398561; doi:10.1371/journal.pone.0175876)
Supplement: S2 Table — This table contains results for fitting of power law distributions to patient co-care networks. (PDF) [file pone.0175876.s008.pdf]

Table S2. Power law best-fit results for patient co-care networks with τ =365 days†

		PLEC		Exponential		Log normal		Weibull		Yule	
	PL-p	LR	p	LR	p	LR	p	LR	p	LR	p
**Provider-Provider**											
Sliding (Full)	0.011	0	1	400	1	0.232	0.984	25.16	1	0.088	0.931
Sliding (Censored)	**0.560**	0	1	365	1	0.018	0.554	11.62	0.980	-0.083	0.425
Binning (Full)	**0.231**	0	1	189	1	0.141	0.971	152	1	0.044	0.935
Binning (Censored)	**0.928**	0	1	227	1	0.029	0.677	8.751	0.971	0.060	0.588
trace-route (Full)	**0.521**	0.036	1	240	1	0.152	0.971	18.83	0.998	0.113	0.946
trace-route (Censored)	**0.301**	0	1	260	1	0.076	0.973	10.56	0.992	1.260	0.92
**Org-Org**											
Sliding (Full)	0	**-26.66**	<0.001	0.767	0.522	-24.55	<0.001	-24.89	<0.001	-0.110	<0.001
Sliding (Censored)	0	**-15.64**	<0.001	56.49	1	-12.85	0.001	-13.83	0.001	-0.658	<0.001
Binning (Full)	0	**-330**	<0.001	3500	1	-192	<0.001	-219	<0.001	-3.965	<0.001
Binning (Censored)	0	**-399**	<0.001	-230	<0.001	8731	1	-210	<0.001	-63.35	<0.001
trace-route (Full)	0.043	**-9.10**	<0.001	6.474	0.756	-7.911	0.007	-7.488	0.059	-0.046	<0.001
trace-route (Censored)	0.252	**-7.18**	<0.001	23.38	0.982	-5.568	0.016	-6.109	0.015	-0.339	<0.001

PL-p: Power Law p-value Calculated by fitting 1,000 random power law curves, where the null hypothesis is that the data adheres to power law, LR: Likelihood Ratio, p: p-value (Whether distribution is a better than a power law), PLEC: Power law with exponential cutoff. Statistically significant values are in bold face type.
†Algorithm explicitly excludes self-loops. ‡Metrics for undirected graph.
