# Supplementary material for: Properties of healthcare teaming networks as a function of network construction algorithms
Source: PLoS One. 2017 Apr 20;12(4):e0175876. doi: 10.1371/journal.pone.0175876 (PMC5398561; doi:10.1371/journal.pone.0175876)
Supplement: S3 Table — This table has shows the fraction reduction in edges and nodes as a function of thresholding the edge weights. (PDF) [file pone.0175876.s009.pdf]

Table S3. Edges and nodes after thresholding by edge weight

Threshold	Trace-route		Binning		Sliding Window	
	E (%)	V (%)	E (%)	V (%)	E (%)	V(%))
1	100.0	100.0	100.0	100.0	100.0	100.0
2	73.8	28.0	72.49	27.3	96.1	64.4
3	65.3	14.5	61.0	14.8	91.7	46.0
4	60.4	9.3	53.8	9.7	88.2	36.6
5	56.8	6.7	48.4	7.0	84.7	29.5
6	54.1	5.2	44.2	5.4	82.2	25.5
7	51.7	4.2	40.7	4.3	79.3	21.6
8	49.7	3.5	37.8	3.5	77.3	19.3
9	47.9	3.0	35.3	2.9	74.9	17.0
10	46.4	2.6	33.1	2.5	73.0	15.3
11	44.9	2.3	31.2	2.1	70.8	13.8
